# Lingual Foramina Anatomy: An Observational Study in Dry Mandibles

**DOI:** 10.3390/dj13050218

**Published:** 2025-05-19

**Authors:** Charalambos Tsatsarelis, Zoi Maria Thomaidi, Vasileios Papadopoulos

**Affiliations:** Laboratory of Anatomy, Department of Medicine, Democritus University of Thrace, 67100 Alexandroupolis, Greece; chartsat2@med.duth.gr (C.T.); zt221487@students.euc.ac.cy (Z.M.T.)

**Keywords:** mandible, lingual foramen, genial tubercle, alveolar process, alveolar ridge

## Abstract

**Background/Objectives**: The lingual foramina of the mandible serve as passageways for arterial branches that are susceptible to injury during surgical procedures, potentially leading to varying degrees of hemorrhage. The objective of the present study was to contribute to the quantification and classification of lingual foramina using cadaveric dry mandibles in relation to surgical safety and, especially, to the risk of perioperative bleeding. **Methods**: This study examined the number, diameter, and spatial relationship of lingual foramina to the genial tubercle, alveolar process, and alveolar crest in dry mandibles. Stainless steel wire threads and Digimatic caliper measurements were utilized. Cluster analysis was employed for the classification of foramina into distinct spatial groups. One-way analysis of variance (ANOVA) was used to compare mean values among ≥3 groups. **Results**: A total of 100 dry mandibles were initially analyzed for the presence of lingual foramina with a diameter of ≥2 mm. In 96 of them (50 dentate and 46 edentulous), 387 lingual foramina (mean: 4.03 per mandible) were recognized; the remaining 4 had smaller lingual foramina (diameter <2 mm). Only 4 mandibles (4.2%) exhibited a single lingual foramen, whereas the remaining 92 (95.8%) displayed multiple foramina (up to nine). The observed lingual foramina had a diameter of 0.44 ± 0.02 mm and were located at distances of 8.74 ± 0.54 mm from the genial tubercle, 14.19 ± 0.87 mm from the alveolar crest, and 14.53 ± 0.84 mm from the inferior border of the mandible. Based on their relationship to the genial tubercle, the foramina were classified into four distinct groups: (i) right (27/387—7%), (ii) proximal (254/387—66%), (iii) superior (81/387—21%), and (iv) left (25/387—6%). The superior group exhibited the largest mean diameter (0.52 ± 0.22 mm, ANOVA *p* < 0.001). The probability of detecting a lingual foramen was minimized at a distance of 13.00 ± 0.50 mm from the genial tubercle, delineating a relatively safe zone with a lower risk of hemorrhage. **Conclusions**: This study provides anatomical insights that contribute to appropriate preoperative planning and the minimization of complications during surgical interventions on the mandible.

## 1. Introduction

The mandible has an intricate internal structure. Within it lies a network of canals that accommodate arteries, veins, and nerves, forming the vascular and nervous plexuses that supply both the teeth and the mandible itself. These vessels originate from major arteries such as the sublingual, subgenial, and mylohyoid arteries, entering the mandible through foramina located on its posterior surface, typically between the first premolars. These openings are collectively known as the lingual foramina.

Two groups of lingual foramina have been described in relation to their location in the mandible, namely, the median lingual foramina (MLF) and the lateral lingual foramina (LLF). Furthermore, the MLF have been further categorized as superior (s-MLF) and inferior (i-MLF) in relation to the genial tubercle. The associated to a lingual foramen vascular canal is called lingual vascular canal (LC); in keeping with the terminology proposed for lingual foramina, an LC could be either median (MVC) or lateral (LVC) [1].

Lingual foramina have been widely studied due to their clinical significance, particularly the medial lingual foramen, which is positioned above the genial tubercle. While most research has focused on this foramen, smaller foramina in the interpremolar region have received less attention. This may be due to their diminutive size, which makes them more difficult to detect, especially in cone-beam computed tomography (CBCT) scans, where smaller foramina may go unnoticed. Consequently, their presence and clinical importance are often underestimated [1,2,3].

Arterial branches pass through the lingual foramina, either entering or exiting the mandibular cortex. The diameters of these branches range from 0.2 mm to 1 mm, reflecting the size of the accompanying neurovascular bundle. During surgical procedures in this region, these vessels are at risk of injury, which can lead to varying degrees of hemorrhage. Severe bleeding may result in a hematoma capable of obstructing the airway, while less significant hemorrhage can compress nearby nerves, causing ischemia and sensory deficits in the mandibular teeth or gingivae [1].

In this observational study, we analyzed the number and diameter of lingual foramina in dry mandibles housed in the osteological collection of the Laboratory of Anatomy, Department of Medicine, Democritus University of Thrace, Greece, aiming to contribute to the quantification and classification of lingual foramina using cadaveric dry mandibles in relation to surgical safety and, especially, to the risk of perioperative bleeding. In detail, we aimed to map the foramina relative to specific anatomical landmarks, including the genial tubercle, alveolar process, and alveolar ridge, and examined the positioning of the genial tubercle on the posterior surface of the mandible. Moreover, we aimed to identify a potential bleeding risk zone, thus providing valuable anatomical insights for surgeons and aiding in preoperative planning to minimize complications and reduce the risk of patient mortality.

## 2. Materials and Methods

### 2.1. Specimen Selection

The present observational study examined 100 dry mandibles from the Laboratory of Anatomy, Department of Medicine, Democritus University of Thrace, Greece. The mandibles originated from an undefined population of Greek ancestry, with no available data on age or sex. All dentate mandibles had at least one well-defined alveolus. Mandibles exhibiting fractures, erosion, or deformities were excluded from the study. The study focused on determining the number, diameter, and anatomical position of lingual foramina relative to the genial tubercle and alveolar process. Additionally, the position of the genial tubercle on the posterior surface of the mandible was assessed.

The quality of the present study, based on the potential risk of bias due to the methods used and the reporting of results, was judged using the Anatomical Quality Assessment (AQUA) tool [4].

An ethics committee approval was not applicable due to the cadaveric material studied.

### 2.2. Measurements

The study objectives were to measure the number, location, and diameter of lingual foramina as well as the distance between lingual foramina and the genial tubercle, alveolar ridge/superior border, and inferior border.

The foramina located on the posterior surface of the mandible between the first premolars were included in the analysis. The number of lingual foramina was determined through careful inspection of the lingual surface under natural daylight. Their diameters were measured using flexible stainless-steel wires with a diameter increasing from 0.2 mm to 1.2 mm, in steps of 0.1 mm (UA218893; Zhejiang, China), as described elsewhere [5]. Any foramen with a diameter smaller than 0.2 mm was not recorded. If a wire could not be inserted into a foramen, its diameter (d_i_) was recorded as the next smallest available size. As such, the absolute maximum error (d_e_) introduced in each measurement is 0.1 mm, while the mean relative error is represented by the mean of the ratios d_e_/(d_i_ + d_e_) for every i-th foramen.

All measurements were performed using a Digimatic caliper (Mitutoyo Co., Kawasaki, Kanagawa, Japan) of 0.01 mm accuracy.

**Figure 1:**
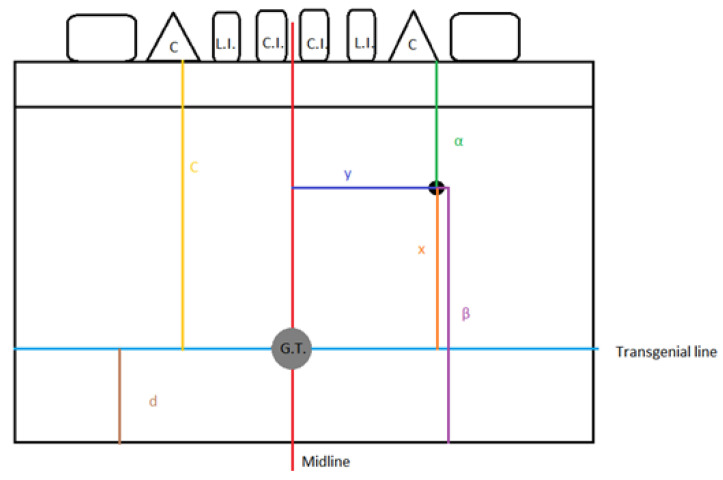
Measurements protocol; x: height; y: length; α: foramen–alveolar ridge/superior border distance; β: foramen–inferior border distance; c: genial tubercle–alveolar ridge/superior border distance; d: genial tubercle–inferior border distance.

Each distance was measured from the center of each lingual foramen. The location of each lingual foramen was determined using two imaginary lines (horizontal and vertical) intersecting at the genial tubercle, dividing the area into four quadrants: anterior superior, posterior superior, anterior inferior, and posterior inferior. In detail, to determine the position of each foramen, the following distances were measured: (i) Height, namely, the vertical distance between the genial tubercle and the foramen (x); (ii) Length, namely, the horizontal distance between the genial tubercle and the foramen (y); (iii) Radius, namely, the distance between the genial tubercle and the foramen (represented by the square root of x^2^ + y^2^); (iv) Distance from the foramen to the alveolar ridge/superior border (α); and (v) Distance from the foramen to the inferior border (β). Similarly, to determine the position of the genial tubercle on the posterior surface of the mandible, the following measurements were taken: (v) Distance from the genial tubercle to the alveolar ridge/superior border (c); Distance from the genial tubercle to the inferior border (d) (Figure 1).

We defined the transgenial line as the horizontal line that traverses the middle of the genial tubercle. The morphology of the tubercle does not have an impact on the position of the line.

In dentate mandibles, the superior margin of the alveolar process was used as a reference landmark. In contrast, for edentulous mandibles, the superior border of the mandible served as the reference for determining the positions of the lingual foramina and genial tubercle. All measurements were compared between dentate and edentulous mandibles.

### 2.3. Quality Assessment

Using the Anatomical Quality Assessment (AQUA) tool, we assigned a high risk of bias to Domain 1 (Objective(s) and subject characteristics) due to the lack of information regarding sex, age, and medical history of the cadavers. On the contrary, all other Domains, namely Domain 2 (Study Design), Domain 3 (Methodology Characterization), Domain 4 (Descriptive Anatomy), and Domain 5 (Reporting of Results) were considered as having low risk of introducing bias.

### 2.4. Statistics

Continuous variables were expressed as means and standard errors (SE) and compared using Student’s *t*-test in cases where equal variances could be assumed; otherwise, Welch’s *t*-test was alternatively preferred. Discrete variables were expressed as percentages and compared using chi-square tests; in cases of expected frequencies <5 in ≥25% of cells, Fisher’s exact test was applied. The chi-square test was performed to evaluate the goodness-of-fit in the quadrants. ANOVA along with Tukey’s HSD posthoc test were implemented to compare means along clusters. Two-step cluster analysis was further used to discriminate lingual foramina according to their direction and distance from the genial tubercle. The level of statistical significance was set to 0.05. All tests were two-tailed and performed using SPSS 26.0 [6].

## 3. Results

### 3.1. Primary Data

**Figure 2:**
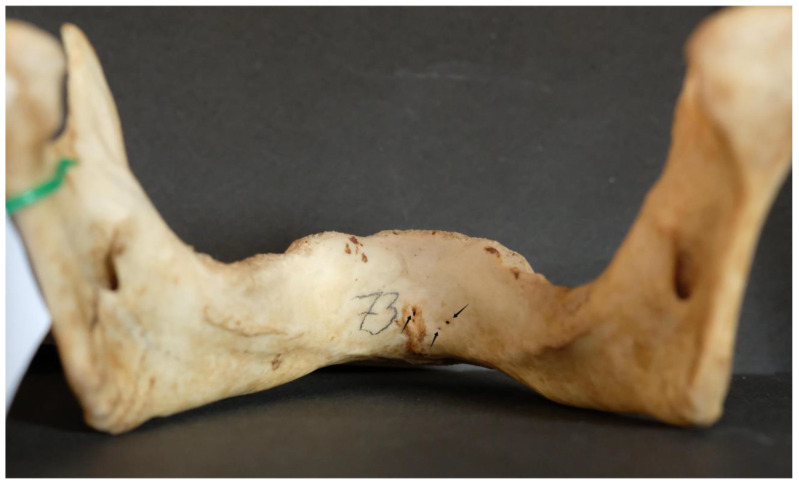
A typical dry mandible; lingual foramina are indicated by arrows.

A total of 100 dry mandibles were initially analyzed for the presence of lingual foramina with diameters of ≥2 mm. Four mandibles had smaller lingual foramina (diameter < 2 mm) and were not considered in further analysis. In the remaining 96 mandibles (50 dentate and 46 edentulous), 387 lingual foramina (mean: 4.03 per mandible) were recognized; in these mandibles, the genial tubercle was found to be located 15.27 ± 1.11 mm from alveolar ridge and 14.42 ± 0.37 from inferior border (Figure 2).

**Figure 3:**
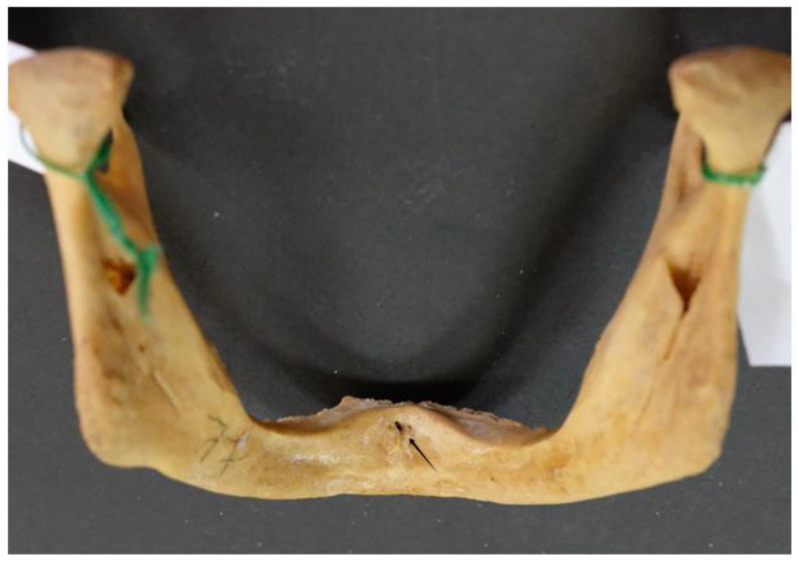
Single lingual foramen (arrow).

Out of the 96 mandibles analyzed, 4 (4.2%) had a single lingual foramen (Figure 3).

**Figure 4:**
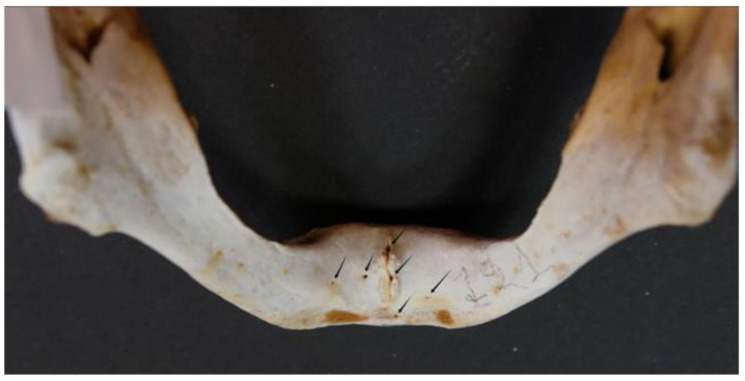
Multiple (six) lingual foramina indicated by arrows.

**Figure 5:**
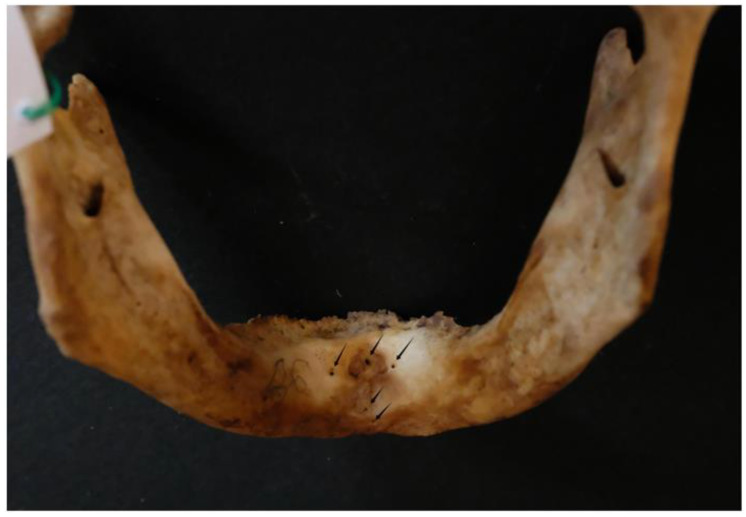
Multiple (five) lingual foramina indicated by arrows.

The majority of the mandibles examined (92; 95.8% of the sample) had multiple lingual foramina (up to nine) (Figure 4 and Figure 5).

**Figure 6:**
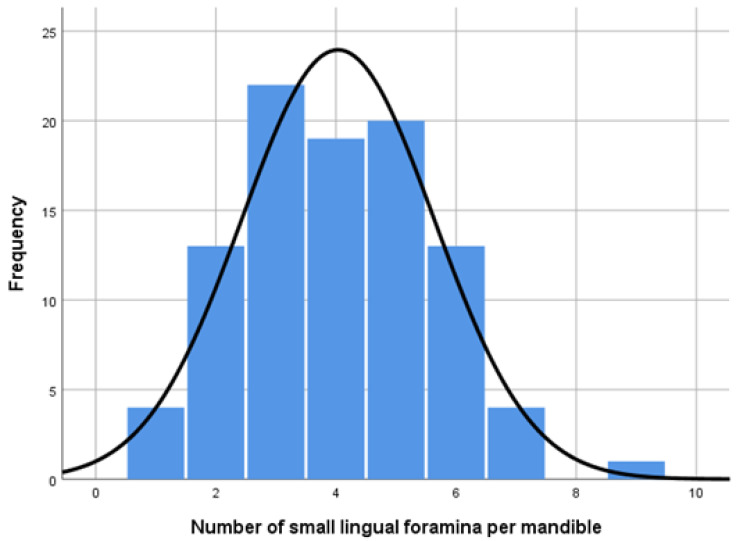
Histogram depicting the number of lingual foramina per mandible in the study sample; the y-axis represents count.

The distribution of the number of lingual foramina among the mandibles examined is depicted in Figure 6.

The lingual foramina observed varied in size, ranging from 0.2 to 1.5 mm.

The average diameter of the foramina was 0.44 ± 0.02 mm; the mean value of the maximum relative error of these measurements was 0.210 (95% CI: 0.202–0.218), thus introducing a mean bias of 21%.

The average location of the foramina was 8.74 ± 0.54 mm from the genial tubercle (height 7.57 ± 0.56 mm; length 4.24 ± 0.54 mm), 14.19 ± 0.87 mm from the alveolar ridge, and 14.53 ± 0.84 mm from the inferior border of the mandible.

The lingual foramina observed were located mostly in the left hemimandible (left vs. right: 218 vs. 169; *p* = 0.013); however, small lingual foramina were comparable when the level of the genial tubercle was considered (upwards vs. downwards: 196 vs. 191; *p* = 0.799).

Taken together, small lingual foramina were more prevalent in the upper left quadrant, in relation with the genial tubercle (*p* = 0.043) (Table 1).

Overall, the 25%, 50%, 75%, and 90% of lingual foramina could be traced at distances of 4.56, 9.03, 16.56, and 20.86 mm, respectively; these distances vary according to the quadrant (Table 2).

### 3.2. Clustering Small Lingual Foramina Reveals a Lower Bleeding Risk Zone

Having detected significant differences in the distribution of lingual foramina in quadrants, we aimed to further analyze the special infrastructure of lingual foramina, thus delineating any potential subgroups among MLF and LLF and investigating their characteristics.

**Figure 7:**
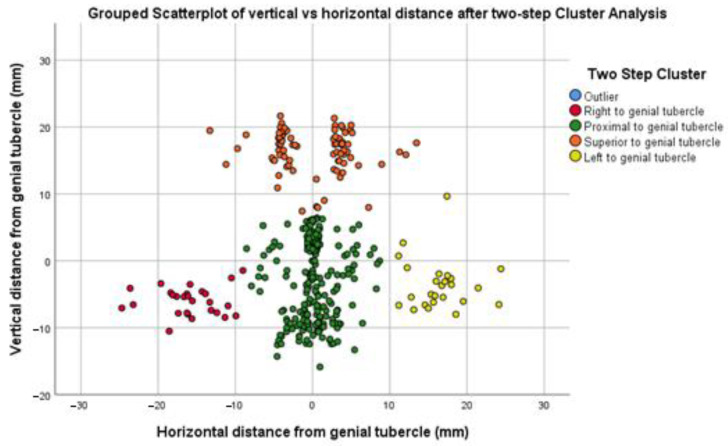
Grouped scattergram depicting vertical vs. horizontal distance of small lingual foramina from genial tubercle after two-step cluster analysis: four discrete clusters determine a relatively safe, lower “bleeding risk zone” amongst them.

For that purpose, a grouped scattergram depicting the vertical vs. horizontal distance of small lingual foramina from the genial tubercle is provided as Figure 7. This scattergram visualizes the results of a two-step cluster analysis, showing four distinct groups of data points corresponding to the 387 small lingual foramina recorded: (i) right ofthe genial tubercle (from now on called R-LLF), (ii) proximal to the genial tubercle (from now on called P-MLF), (iii) superior to the genial tubercle (S-MLF), and (iv) left ofgenial tubercle (from now on called L-LLF).

**Figure 8:**
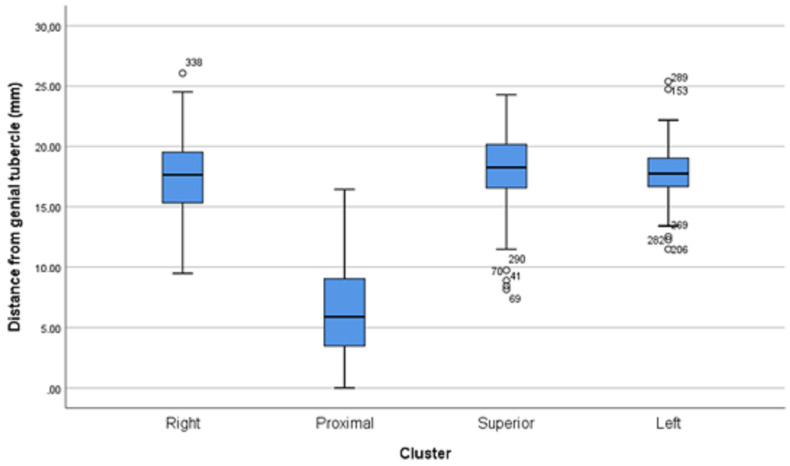
Distance from genial tubercle per cluster.

Distances from the genial tubercle are provided in Figure 8.

In detail, the characteristics of small lingual foramina per cluster are shown in Table 3. The small lingual foramina of the superior cluster are significantly larger when compared with the foramina of the right (*p* = 0.008), proximal (*p*< 0.001), and left (*p* = 0.022) clusters.

Of note, 6/387 (1.8 %) foramina presented a diameter ≥ 1.0 mm (3 proximal; 3 superior.

**Figure 9:**
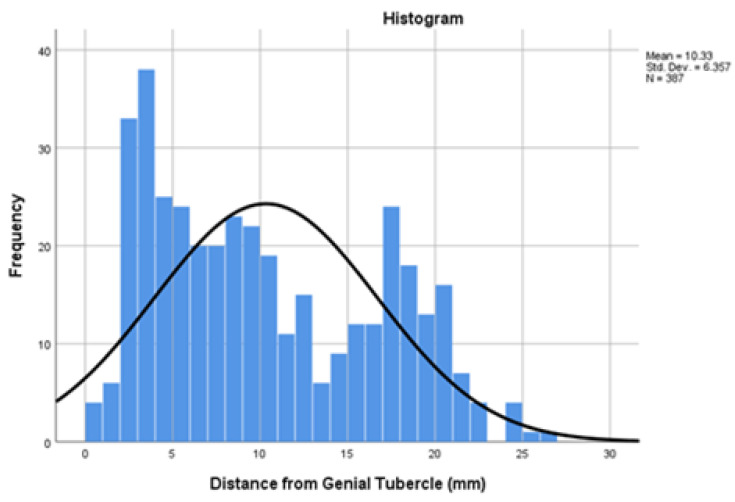
Histogram depicting bimodal distribution of foramen distances from genial tubercle; the y-axis represents count.

Of note, a bimodal distribution of foramen distances from the genial tubercle is observed. The first mode is between 3.00–4.00 mm, corresponding to the proximal cluster. The second mode is between 17.00–18.00 mm, corresponding to the right, superior, and left clusters. An interstitial, relatively safe “bleeding risk zone” lies between 13.00 ± 0.50 mm (Figure 9).

### 3.3. Differences Between Dentate and Edentulous Mandibles

A detailed comparison between dentate and edentulous mandibles is provided in Table 4.

**Figure 10:**
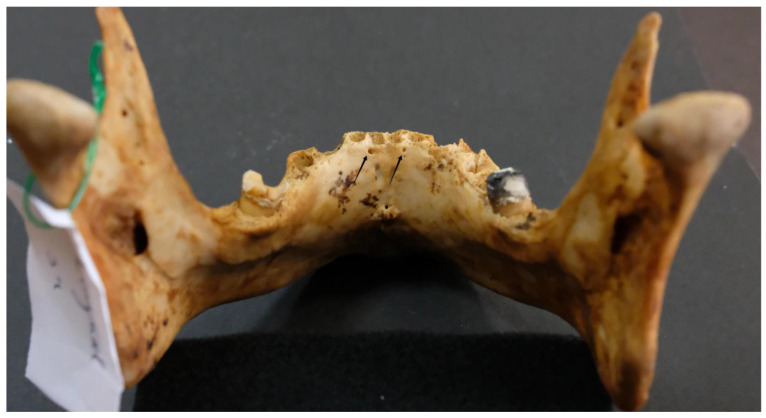
Lingual foramen detected in alveolar position (arrows).

Lingual foramina that were close to the alveolar ridge (Figure 10) were usually more detected in dentate mandibles, when compared to edentulous ones.

**Figure 11:**
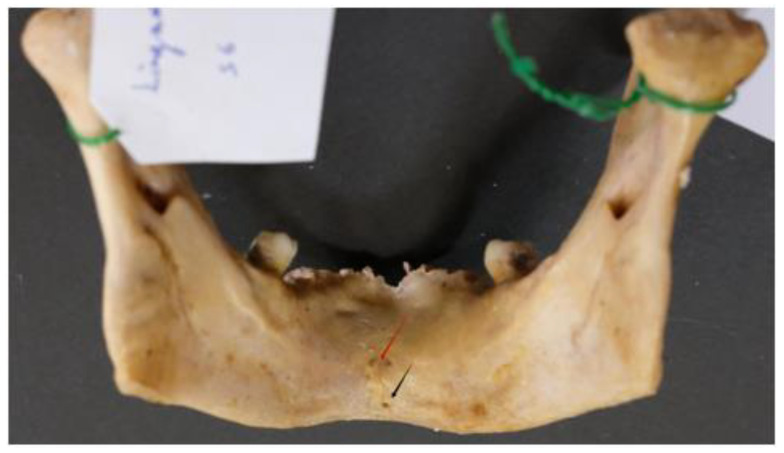
Mandible lacking central genial lingual foramina (arrows).

Interestingly, only one mandible had no central genial lingual foramen (Figure 11).

## 4. Discussion

The present observational study included a detailed investigation of all lingual foramina of diameters ≥ 0.2 mm in dry mandibles using stainless-steel wires; to the best of our knowledge, this is the first time that this approach has beenfollowed in cadaveric material.

We have focused on cadaveric material, instead of CBCT, for two reasons: First, the CBCT has a lower ability to detect small lingual foramina; second, a lingual duct might be larger than the opening of the related lingual foramen, and thus, more clinically significant. Interestingly though, our results concerning the number of the lingual foramina recognized per mandible (4.03) are in keeping with recent CBCT-derived data [7].

In their meta-analysis, Bernardi et al. reported that lingual foramina diameters from cadaveric and radiologicalCBCTare comparable; however, the pooled mean diameter is almost double that of the present study (0.84 mm vs. 0.44 mm). This could be attributed to the different method used [8]. Presumably, small foramina (<3 mm in diameter) are hardly detectable by CBCT, though easily investigated in cadaveric material; therefore, the preoperative evaluation by CBCT may miss the presence of these foramina, thus underestimating the bleeding risk.

Silvestri et al. reported that the number, diameter, length, and orientation of foramina do not appear to be altered when a patient is edentulous. On the contrary, we have detected significant differences between edentulous and dentate mandibles as far as their distances from the genial tubercle, alveolar ridge, and inferior border areconcerned [3].

Although large lingual foramina (≥1.0 mm) are rare, their presence near the midline poses a risk of both vascular and neural injury during surgery. Therefore, it is essential to consider lingual foramina during surgical procedures involving the anterior mandibular region. These additional openings in the lingual surface of the mandible host nerves and blood vessels [9]. Damaging of this neurovascular bundle during surgery can result in neurosensory disturbances of the mandibular teeth or bleeding.

Understanding the location of the lingual foramina is crucial when performing anterior mandible surgery. The lingual foramina are at risk in many dental and oral surgery procedures at the mental region such periapical surgery, genioplasty, implant placement, treatment of symphyseal fractures, preprosthetic and grafting procedures, and the removal of cysts or pathologic lesions [1,10,11]. The role and the contribution of these accessory foramina to regional tumorspread has also been documented [12,13].

Oral implants are routinely employed in the rehabilitation of the edentulous mandible, with symphyseal implant placement frequently considered a relatively uncomplicated procedure. Despite this assumption, severe complications from implant surgeries in this region are a documented reality. The lingual foramina serve as a conduit for branches of the sublingual and submental arteries, facilitating anastomosis with the central inferior alveolar vessels. Damage to these vessels can lead to significant bleeding, potentially forming a sublingual hematoma in the floor of the mouth. In severe cases, the hematoma can expand, causing swelling that can obstruct the airway [14,15,16]. Hospitalization may be necessary for monitoring and treatment. Severe bleeding can require emergency intervention, including airway management (e.g., intubation or tracheostomy) [17,18,19,20,21,22].

Thorough preoperative imaging, such as CBCT, is crucial. CBCT can help identify the location and size of lingual foramina, allowing for careful surgical planning. Although serious complications are relatively rare, clinicians need to be well prepared to deal with them.

In this study, we assessed lingual foramina locations by measuring distances from the lingual foramina to both the alveolar crest and the inferior border of the mandible. The latter measurement is considered more reliable, as it remains consistent regardless of whether the mandible is dentate or edentulous. In contrast, the distance between the lingual foramina and the alveolar ridge can change due to resorption of the alveolar ridge with age [23]. Alveolar ridge resorption in edentulous mandibles significantly alters the location of lingual foramina [24,25]. As the alveolar bone resorbs, especially in advanced stages, the medial lingual foramina can “shift” in position. This anatomical change increases the risk of vascular perforation and hematoma formation during drilling, even when short implants are selected. However, several studies have demonstrated the superior accuracy and reliability of CBCT when compared to the conventional imaging techniques for identifying small foramina as accessory mental foramina [26]. This enhanced visualization makes CBCT an invaluable tool for presurgical planning and execution, enabling clinicians to minimize potential complications and achieve optimal results.

The major strength of this study is the use of cluster analysis to discriminate subgroups of lingual foramina and investigate their characteristics. To our knowledge, this is the first study of its kind. As such, it is novel and adds to the already established literature. On the contrary, one couldargue that the use of a not widely utilized method, namely, that of the stainless-steel wires, to determine the diameter of foramina, could be a relative limitation. Indeed, this method introduces a bias, as the measured diameter is always less than the actual diameter. However, the extent of this discrepancy, namely, the absolute error (d_e_), remains below 0.1 mm. The absolute error could be further limited if the diameters of the wires used increased in smaller steps (e.g., 0.05 mm). Moreover, the intraclass correlation coefficient estimates of this method, based on the mean rating of two independent observers, is very close to absolute (0.987–1.000), as analyzed elsewhere [5] Additionally, the data regarding the prevalence of rare anatomic variants, such as multiple (>8) lingual foramina, or the absence of a central lingual foramen, might be considered prone to considerable reporting bias [27]. Lastly, the lack of age- and sex-specific data could be considered as a certain limitation, as it might conceal any potentially attributable differences [28]. Of note, this deficit is usual on cadaveric studies [2]. However, the interpretation of anatomical variability might not be largely affected, as data derived from CBCT scans of edentulous patients found no significant sex- or age-based differences in the number of foramina [3]. The unavailability of direct comparison of our cadaveric material with corresponding CBCT data constitutes another limitation. Future research could further enlighten the clinical impact of the present anatomical results.

## 5. Conclusions

This study provides anatomical insights that contribute to appropriate preoperative planning and the minimization of complications during surgical interventions on the mandible.

## Figures and Tables

**Table 1:** Goodness-of-fit chi-square test between frequencies in quadrants.

Foramina Location	n (%)	*p*-Value
Left superior quadrant	117 (30.2)	0.043
Right superior quadrant	79 (20.4)	
Left inferior quadrant	101 (26.1)	
Right inferior quadrant	90 (23.3)	

**Table 2:** Distances in mm from genial tubercle that contain lingual foramina at a given probability/odds ratio; data for all (2nd column), above genial tubercle (3rd column), and below genial tubercle (4th column).

Probability/OR for a Foramen to Be Included in the Zone	All Lingual Foramina; mm (n = 387)	Lingual Foramina Above Genial Tubercle; mm (n = 196)	Lingual Foramina Below Genial Tubercle; mm (n = 191)
25%/0.33	4.56	5.45	3.69
50%/1.00	9.03	8.20	5.93
75%/3.00	16.56	10.03	17.22
95%/19.00	20.86	12.73	20.56

**Table 3:** Small lingual foramina characteristics per cluster.

	Right (R-LLF) (n = 27)	Proximal (P-MLF) (n = 254)	Superior (S-MLF) (n = 81)	Left (L-LLF) (n = 25)	*p*-Value
Diameter	0.39 ± 0.10	0.42 ± 0.19	0.52 ± 0.22	0.40 ± 0.16	<0.001
Height	6.41 ± 2.10	5.61 ± 3.52	16.97 ± 3.12	5.01 ± 2.39	<0.001
Length	16.26 ± 3.99	2.18 ± 2.02	4.89 ± 2.55	16.82 ± 3.52	<0.001
Distance from genial tubercle	17.64 ± 3.81	6.43 ± 3.67	17.83 ± 3.24	17.72 ± 3.48	<0.001
Distance from alveolar ridge	22.88 ± 5.70	16.62 ± 7.13	4.12 ± 4.61	19.50 ± 6.37	<0.001
Distance from inferior border	9.71 ± 2.37	11.90 ± 5.39	27.79 ± 5.35	10.21 ± 3.84	<0.001

**Table 4:** Descriptive statistics and comparison between dentate and edentulous mandibles; values are expressed in mm.

Parameter	All Mandibles (n = 96)	Dentate Mandibles (n = 50)	Edentulous Mandibles (n = 46)	*p*-Value
**Foramina number**				
Per mandible **(n)**	4.03 ± 0.16	4.54 ± 0.23	3.48 ± 0.20	0.001
**Foramina location** (n)				
Right cluster (R-LLF)	27	20	7	<0.001
Proximal cluster (P-MLF)	254	122	132	
Superior cluster (S-MLF)	81	69	12	
Left cluster (L-LLF)	25	16	9	
**Foramina characteristics** (mean ± SE)				
Diameter	0.44 ± 0.02	0.45 ± 0.01	0.43 ± 0.02	0.094
Height	7.57 ± 0.56	8.81 ± 0.42	5.80 ± 0.30	<0.001
Length	4.24 ± 0.54	5.03 ± 0.39	3.11 ± 0.34	0.002
Distance from genial tubercle	8.74 ± 0.54	12.02 ± 0.46	7.93 ± 0.36	<0.001
Distance from alveolar ridge	14.19 ± 0.87	15.46 ± 0.64	12.38 ± 0.53	<0.001
Distance from inferior border	14.53 ± 0.84	16.79 ± 0.59	11.31 ± 0.48	<0.001

## Data Availability

All data used can be provided upon reasonable request.
